# Colorectal cancer risk in association with colorectal cancer as a second malignancy in relatives: a nationwide cohort study

**DOI:** 10.1186/s12885-022-10000-z

**Published:** 2022-08-18

**Authors:** Guoqiao Zheng, Jan Sundquist, Kristina Sundquist, Jianguang Ji

**Affiliations:** 1 Center for Primary Health Care Research, Lund University/Region Skåne, Jan Waldenströms gata 35, 205 02 Malmö, Sweden; 2 Department of Family Medicine and Community Health, Department of Population Health Science and Policy, Icahn School of Medicine at Mount Sinai, New York, USA; 3 Center for Community-based Healthcare Research and Education (CoHRE), Department of Functional Pathology, School of Medicine, Shimane University, Matsue, Japan

**Keywords:** Colon cancer, Rectal cancer, Familial clustering, Multiple primary cancer

## Abstract

**Background:**

Increasing number of individuals will have first-degree relatives (FDRs) diagnosed with colorectal cancer (CRC), as a second primary malignancy (CRCa-2) after a non-CRC cancer. We aimed to estimate whether and to what extent a family history of CRCa-2 is associated with an increased CRC risk.

**Methods:**

In this Swedish nationwide cohort study, rate ratio (RR) and cumulative incidence of CRC were estimated among 172,531 individuals with a family history of CRC as a first primary malignancy (CRCa-1) and 17,830 with a family history of CRCa-2, respectively, using individuals without cancer family history as the reference group.

**Results:**

A cumulative incidence of CRC by age 80 was 6.3 and 5.6% for individuals with a parental and a sibling family history of CRCa-2, respectively. RRs of CRC for one FDR diagnosed with CRCa-1 and CRCa-2 were respectively 1.72 (95% CI, 1.65–1.79) and 1.50 (1.32–1.70); the latter RR was lower than the former (*P* = 0.0356), but no difference was observed after adjusting age of diagnosis of CRC in FDR and family relationship (*P* = 0.6898). Increased RRs were found to be associated with a CRCa-2 diagnosis in FDR that occured after cancers in upper aerodigestive tract, breast, prostate, kidney and nervous system.

**Conclusions:**

Individuals who have relatives with CRCa-2 have an increased risk of CRC, but the magnitude is lower than those having relatives with CRCa-1, which is related to different ages of diagnosis of CRC in FDR and family relationships.

**Supplementary Information:**

The online version contains supplementary material available at 10.1186/s12885-022-10000-z.

## Introduction

Colorectal cancer (CRC) ranks third among all cancers and is the leading cause of cancer-related death worldwide [1]. Largely due to the implemented CRC screening, the incidence rate of CRC has started to stabilize or decrease in high-income countries [2]. However, evidence shows that the incidence rate of CRC is rising among those younger than 50 years old) [3]. Thus, a more comprehensive screening strategy is required as the rising incidence rate of CRC in young adults reflects the future disease burden. Recently, it has been proposed that the starting age of CRC screening should be adapted in accordance with the individual familial risk [4]. The lifetime risk of CRC for individuals having one parent and sibling with CRC was estimated to be 7 and 6.5%, which indicates a 1.7 and 1.65-fold increase over the risk in those without any family history, respectively [5]. The familial risk varies according to the number of affected relatives, age at diagnosis of CRC in relatives, and the familial relationship (for example, parents-offspring or siblings) [6]. However, almost none of the previous studies on familial clustering of CRC distinguished the order of the CRC diagnosis (CRC as a first primary malignancy, CRCa-1, or CRC as a second primary malignancy, CRCa-2) in relatives. On the other hand, because of the improvements in cancer treatment and the enforcement of cancer screening, more and more cancer survivors might develop second primary cancer [7]. The cumulative incidence of a second primary cancer ranges from 2 to 17% among cancer survivors [8–10]. Unfortunately, it is still unknown if the risk of CRC among individuals whose first-degree relatives (FDRs, parents or siblings) were diagnosed with CRCa-2 will be increased similarly as those with a family history of CRCa-1. The risk factors that increase the occurrence of CRCa-1 can also predispose CRCa-2. On the other hand, the development of CRCa-2 can also be induced by the treatment for first primary cancer, which cannot be shared among family members. Furthermore, there may be difference in age at diagnosis of CRCa-1 and CRCa-2, and early-onset CRC is associated with relative high prevalence of hereditary cancer syndromes and familial component [11]. Therefore, we expect a different familial risk associated with a family history of CRCa-2. The aims of our study were to estimate whether and to what extent a family history of CRCa-2 is associated with an increased risk of CRC.

## Methods

### Data resources

This study was performed by incorporating several Swedish national registers, including the Swedish Multi-Generation Register, Swedish Cancer Register, Cause of Death Register and Total Population Register. The linkage of registers was based on the unique personal identification number that has been replaced by a serial number to preserve confidentiality. All the offspring born after 1931, together with their biological parents, were recorded in the Multi-Generation Register, which enabled us to identify family relationships in FDRs. The Swedish Cancer Register, with national coverage of over 90%, recorded all incident tumors since 1958 [12]. Cancer registration in Sweden is based on compulsory reports of cases diagnosed both by clinicians and by pathologists. A total of two million records of primary invasive cancers were recorded. The notification of cancer was based on the 7th version of the International Classification of Disease (ICD-7) and updated based on subsequent versions. In Sweden, the diagnosis of multiple primary cancers follows the IARC/IACR multiple cancer coding rules [13]. We only considered the CRCs following a first primary non-CRC cancer for CRCa-2 due to the subjective nature of multiple primary diagnosis in the same organ [13]. Information on socioeconomic status, place of residence and death notification were obtained by further linkage to Total Population Register and Cause of Death Register.

### Study population

Family history of CRCa-2 was defined as CRC diagnosis after other first primary cancer in FDRs; in an example shown in Supplementary Fig. 1a, mother was diagnosed with CRC at age2 after cancer A. Family history of CRCa-1 was defined as single CRC diagnosis in FDRs (Supplementary Fig. 1b). Offspring without any cancer diagnosis in FDRs were used as reference group (Supplementary Fig. 1c). The selection of the study population is shown in Supplementary Fig. 2. More than 16 million people were included in this study with national coverage of individuals born after 1931 and their parents. Only offspring generation were included for estimation of the familial risk. The maximum age for the offspring was 84 years of age, whereas the age for the parents was not limited. A total of 8,188,207 individuals in the offspring generation were identified at risk of first primary invasive CRC. We excluded 12,456 offspring who had FDRs diagnosed with multiple primary CRC and 2,361,783 offspring with any FDR affected by any other first primary cancer except those with FDR affected by CRCa-2. Offspring with more than one FDR affected by CRCa-1 or CRCa-2, or those with FDRs affected by higher order primary cancers (such as third, fourth primary cancer) were additionally removed in order to keep the comparison among groups reasonable. Ultimately, the study included 5,595,074 individuals without FDRs affected by any cancer (reference group), 172,531 with a FDR affected by a CRCa-1, and 17,830 with a FDR affected by a CRCa-2. We additionally estimated the risk among 8,186,751 individuals as sensitivity analysis (all the offspring generation except those with a family history of multiple CRC, see Supplementary Fig. 2). The results were stratified based on if other FDRs were diagnosed with cancers other than CRCa-1 and CRCa-2. It should be noted that in this analysis the reference group were individuals without family history of CRC, instead of individuals without family history of cancer.

### Statistical analyses

We employed Poisson regression to compute the rate ratio (RR) of CRC associated with CRCa-2 diagnosed in relatives as well as its two-tailed 95% confidence interval (95% CI). The follow-up started for each individual at birth, immigration date, or 1 January 1958, whichever came latest, and it ended at the date of diagnosis of CRC, death, emigration, or the closing date of the study (31 December 2015), whichever came earliest. The RR was adjusted for age (5-year group), gender, periods (5-year group), socioeconomic status (blue-collar worker, white-collar worker, farmer, private business, professional, or other/unspecified) and place of residence (big cities, northern Sweden, southern Sweden and unspecific). The person-time was apportioned into 5-year attained age bands and 5-year calendar period bands. That means during the follow-up period, the person time of one individual can contribute to different age groups and period groups. The risk was also stratified by gender of the individuals, age at CRC diagnosis in relatives (≤ 60 and > 60 years), family relationship (parent-offspring or siblings), time between first primary cancer and CRCa-2 in relatives (groups based on quartiles) and sites of first primary cancer before CRCa-2 (Supplementary Fig. 2). We further calculated the cumulative incidence and the related 95% CI for CRC from birth to a specific age based on their family history of CRCa-1 or CRCa-2, considering death and diagnosis of other cancer as competing event (R survfit function). We compared the familial risks associated with CRCa-1 and CRCa-2 diagnoses in FDRs in two ways: 1) the full model (the Poisson regression model above), 2) full model with additionally adjusting for age at diagnosis of CRC in FDRs and family relationship. The results could indicate if the possible different familial risk associated with family history of CRCa-1 and CRCa-2 was due to the different age at diagnosis of CRC in FDRs and family relationship. All the statistical analyses were performed with the use of SAS statistical software, version 9.4 (SAS Institute) and R (version 3.6.2).

## Results

At the end of the follow-up, 2769 individuals with a family history of CRCa-1 and 251 with a family history of CRCa-2 were diagnosed with first primary CRC. Compared to FDRs of persons without cancer, the RRs of CRC among offspring with a FDR affected by CRCa-1 and CRCa-2 were 1.72 (95% CI, 1.65–1.79) and 1.50 (1.32–1.70), respectively (See Table 1). The result for the gender stratification showed the similar trend. Early-onset (≤60 years) CRC diagnosis (both CRCa-1 and CRCa-2) in FDR was associated with a higher RR compared to late-onset (> 60 years) diagnosis in FDR, but the difference was not significant. With a parental family history of CRCa-1, the risk was 1.68 (1.60–1.75) and with a sibling family history the risk increased to 1.89 (1.74–2.05). While for CRCa-2, the CRC risks were similarly associated with a parental (RR, 1.51, 1.31–1.73) and sibling (1.45, 1.09–1.93) family history. For the comparison between family history CRCa-1 and CRCa-2, the familial risk associated with CRCa-1 in FDR was higher than that with CRCa-2 in the full model (*P* = 0.0356, Table 1, second last column). However, there was no significant difference after additionally adjusting for the age of CRC diagnosed in FDR and family relationship (*P* = 0.6898, Table 1, last column).

**Table 1:** Colorectal cancer risk among individuals who had a first-degree relative diagnosed with CRCa-1 or CRCa-2

Category		Family history of CRCa-1			Family history of CRCa-2			Comparison ^c^ (CRCa-1 vs. CRCa-2)	
		N ^a^	RR ^b^	95%CI	N ^a^	RR ^b^	95%CI	P1	P2
Overall		2769	1.72	1.65–1.79	251	1.50	1.32–1.70	0.04	0.69
Gender	Among males	1551	1.76	1.67–1.86	143	1.54	1.31–1.83	0.14	0.74
	Among females	1218	1.67	1.58–1.78	108	1.44	1.19–1.74	0.08	0.28
Age of CRC diagnosis in FDR	≤60 years old	536	2.11	1.94–2.30	19	2.16	1.38–3.38	0.93	0.21
	> 60 years old	2233	1.64	1.57–1.72	232	1.46	1.28–1.66	0.08	0.23
Type of family history	Only parent	2147	1.68	1.60–1.75	204	1.51	1.31–1.73	0.15	0.97
	Only sibling	622	1.89	1.74–2.05	47	1.45	1.09–1.93	0.08	0.52

*CRC* Colorectal cancer, *CRCa-1* Colorectal cancer as a first primary malignancy, *CRCa-2* Colorectal cancer as a second primary malignancy

^a^N, number of CRC cases diagnosed during the follow-up

^b^RR was estimated from Poisson regression using individuals without cancer family history as the reference. The covariates adjusted in the model included age groups (5 years), periods (5 years), socioeconomic status (blue-collar worker, white-collar worker, farmer, private business, professional, or other/unspecified) and place of residence (big cities, northern Sweden, southern Sweden and unspecific)

^c^comparison of familial risks associated with family history of CRCa-1 and CRCa-2 with Poisson regression. P1 is the *p* value for the comparison when the adjusted covariates were same as main analysis (above). P2 is the *p* value for the comparison when age at diagnosis of CRC in FDR (as continuous variable) and family relationship were additionally adjusted

**Fig. 1:**
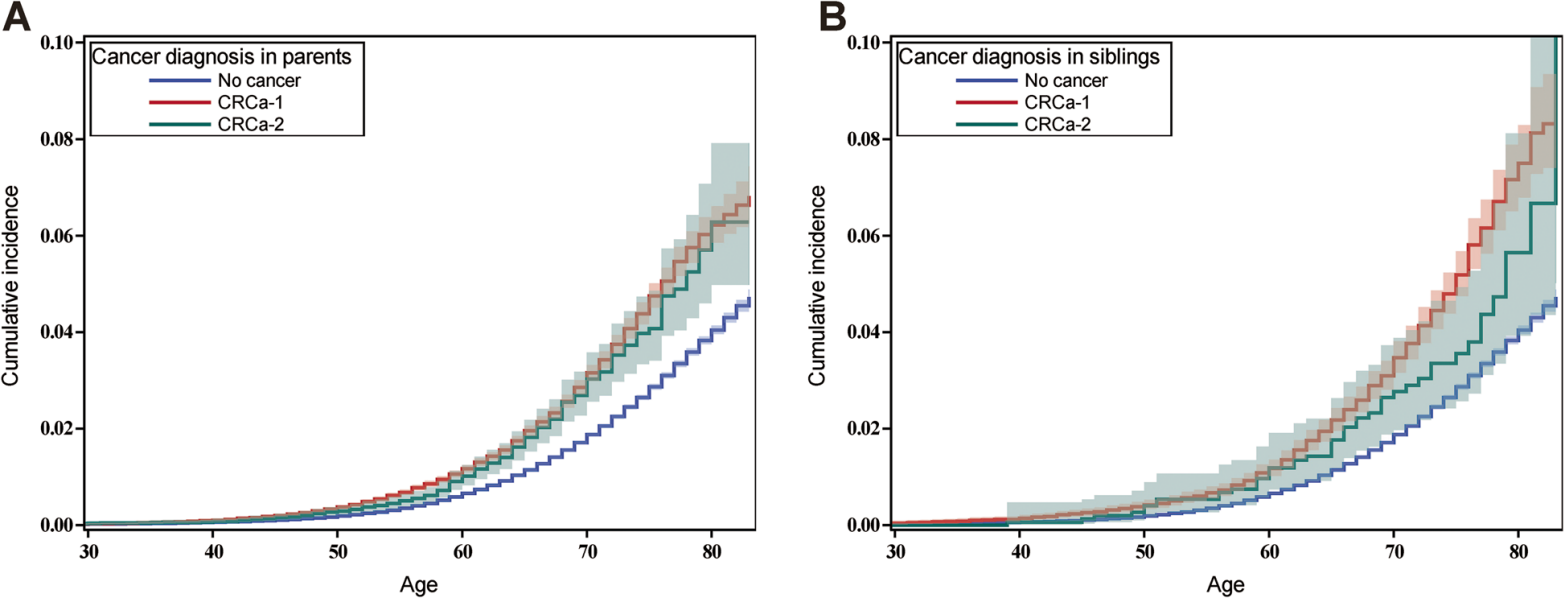
Cumulative incidence of colorectal cancer (CRC) in offspring with family history of CRCa-1 and CRCa-2 in parents (**a**) and siblings (**b**). The shading band is the confidence interval of the cumulative incidence. CRC, colorectal cancer, CRCa-1, colorectal cancer as a first primary malignancy, CRCa-2, colorectal cancer as a second primary malignancy

The cumulative incidence of CRC in offspring stratified by family relationship is presented in Fig. 1. The cumulative incidence of CRC by age 80 was 6.2% (95%CI, 5.8–6.6%) and 6.3% (5.0–7.9%) among individuals with a parent affected by CRCa-1 and CRCa-2, respectively. The cumulative incidence by age 80 was 7.5% (6.8–8.3%) among individuals with a sibling family history of CRCa-1 and 5.6% (3.9–8.1%) with CRCa-2.

We explored the familial risk of CRC stratified by anatomical site of first primary cancer in FDR (Table 2). A significant association was found when the first primary cancer in FDR was located in the esophagus (2.05, 1.10–3.80), breast (1.67, 1.22–2.27), prostate (1.50, 1.16–1.93), kidney (2.36, 1.27–4.39) and nervous system (2.71, 1.50–4.89). However, the non-significant association observed in other sites might be due to the small sample size. Among the 11 CRC patients who had FDR diagnosed with first primary nervous system cancer, eight were meningiomas (seven females and one male). The overall median (IQR) time interval between the first primary cancer and CRCa-2 was 72 (25–150) months. No specific trend was observed for CRC risks over different periods when CRCa-2 in FDR was diagnosed after first primary cancer (Supplementary Table 1). For prostate and kidney cancers, significantly high risks were observed in the second quarter.

**Table 2:** Colorectal cancer risk among individuals with a family history of CRCa-2 stratified by different first primary cancer diagnosed in their relatives

First primary cancer site	Relatives with CRCa-2			
	N ^a^	RR ^b^	95%CI	
UAT	10	**2.05**	1.10	3.80
Stomach	6	1.50	0.67	3.35
Small intestine	< 5	0.81	0.11	5.75
Liver	< 5	0.59	0.08	4.21
Pancreas	< 5	0.87	0.12	6.19
Lung	< 5	1.21	0.45	3.22
Breast	40	**1.67**	1.22	2.27
Cervix	10	1.52	0.82	2.83
Endometrium	15	1.46	0.88	2.41
Ovary	8	1.54	0.77	3.08
Female genital	< 5	0.99	0.14	7.06
Prostate	59	**1.50**	1.16	1.93
Testis	< 5	1.67	0.42	6.69
Male genital	< 5	3.51	0.88	14.02
Kidney	10	**2.36**	1.27	4.39
Bladder	15	1.25	0.76	2.08
Melanoma	10	1.22	0.65	2.26
Skin	13	1.20	0.70	2.07
Eye	< 5	2.33	0.58	9.32
Nervous system	11	**2.71**	1.50	4.89
Thyroid	< 5	1.47	0.37	5.87
Endocrine gland	8	1.72	0.86	3.43
Connective tissue	< 5	1.76	0.44	7.05
NHL	6	1.13	0.51	2.53
Hodgkin lymphoma	< 5	3.01	0.75	12.03
Myeloma	< 5	1.84	0.69	4.90
Leukemia	< 5	0.87	0.28	2.70
CUP	< 5	1.43	0.36	5.72

*CRCa-2* Colorectal cancer as a second primary malignancy, *UAT* Upper aerodigestive tract, *NHL* Non-Hodgkin lymphoma, *CUP* Cancer of unknown primary

^a^N, number of CRC cases diagnosed during the follow-up

^b^RR was estimated from Poisson regression using individuals without cancer family history as the reference. The covariates adjusted in the model included age groups (5 years), periods (5 years), socioeconomic status (blue-collar worker, white-collar worker, farmer, private business, professional, or other/unspecified) and place of residence (big cities, northern Sweden, southern Sweden and unspecific)

The CRC risk in association with family history of CRCa-1 and CRCa-2 among all the offspring in the registry is shown in Supplementary Table 2. Individuals with a family history of multiple CRCs have been removed. Results were stratified based on if any other FDR was affected by first primary non-CRC cancer. For individuals without any other FDR affected by first primary non-CRC cancer (left part), the RR for a family history of one CRCa-2 was 1.58 (1.40–1.77) compared to those without family history of CRC. The RR was lower than that for a family history of one CRCa-1 (1.75, 1.68–1.82). Among individuals who had other FDRs affected by first primary non-CRC cancer, the RR was 1.79 (1.61–1.98) for family history of one CRCa-2 and 1.63 (1.56–1.69) for one CRCa-1 (right part). Among individuals who had more than two FDRs were diagnosed with CRCa-2, the risk became mucher higher, particularly for those who had other FDR diagnosed with first primary non-CRC cancer (6.45, 2.90–14.4).

## Discussion

In the Swedish nationwide registers, we showed that the number of people having a family member with CRCa-2 accounted for more than 10% of that with a FDR affected by CRCa-1. With the trend of rising number of CRCa-2 among cancer survivors, the number of individuals whose FDRs are diagnosed with CRCa-2, will continue to increase. Therefore, it is necessary to investigate the magnitude of the CRC risk in association with CRCa-2 diagnosis in FDRs. Our finding showed a moderately increased CRC risk for a CRCa-2 diagnosis in FDR, which was lower than the risk for a CRCa-1 diagnosis in FDR.

The deviation of overall familial risk for family history of CRCa-1 and CRCa-2 indicates that the underlying pathogenesis that drives the CRCa-1 and CRCa-2 might not be totally the same. Firstly, the lower risk for the family history of CRCa-2 can be partly due to the relatively late-onset of CRCa-2 in FDRs. Consistent with the report from SEER, CRCa-2 was diagnosed at a later age compared to CRCa-1 [14]. While early-onset CRC is associated with a relatively high prevalence (ranging from 9 to 26%) of hereditary cancer syndromes and familial components [11]. This theory is further supported by the insignificant results for the comparison between the two types of familial risks after adjusting the age at diagnosis of CRC in FDR and family relationship. Secondly, the development of some CRCa-2 may be related to radiation therapy from the first primary pelvic cancer [15–17]. For example, radiation therapy in prostate cancer patients was associated with 70% higher risk of rectal cancer compared to those with only surgery [18]. Radiation therapy, the effect of which on second primary cancer development may come after several years and sometimes decades [19], can indirectly contribute to the low familial risk associated with family history of CRCa-2, as it is not shared among family members. Cancer treatment could explain the lower familial risk among individuals who had a FDR diagnosed with CRCa-2 after 4 years of primary prostate cancer than individuals who had a FDR diagnosed with CRCa-2 within 4 years after primary prostate cancer. Despite a significant difference in the familial risk for family history of CRCa-1 and CRCa-2, the magnitude of the difference is small, suggesting the effect of age at diagnosis of CRCa-2 and that of the first cancer treatment are both limited. However, a study with a larger sample size is needed to validate the finding.

When stratifying the familial risk by gender of the offspring, the patterns of the risk for family history of CRCa-1 and CRCa-2 were similar. The high familiar risk was found in offspring with FDR affected by CRCa-1 or CRCa-2 at a young age, respectively, which is in line with the statement mentioned above that early-onset CRC is associated with a high familial aggregation [11]. We also found that sibling family history of CRCa-1 was associated with higher CRC risk in offspring compared to parental family history, which is consistent with another study [6]. In contrast, sibling and parental family history of CRCa-2 showed similar associations with CRC risk in offspring. We suggest this is mainly due to the difference in the age at diagnosis of CRCa-1 and CRCa-2 in siblings; and the median age for the former (61) was younger than the latter (68). This can also explain the big different of cumulative incidence of CRC between sibling family history of CRCa-1 and CRCa-2 in Fig. 1.

The first discordant primary cancer diagnosed before the CRCa-2 can provide evidence on the cause of CRCa-2 in cancer survivors as well as the different familial association with CRC in FDRs. All the first primary cancer sites with significant familial risk may have common genetic and lifestyle-related risk factors with CRC, for example, *CHEK2* mutation for breast, prostate and kidney cancers [20], alcohol intake for cancers in the upper aerodigestive tract and breast [21]. Meningioma and colorectal cancer were possibly linked by reproductive related factors [22], as well as other rare dominant genetic predisposition related to the high familial risk. For ovarian and endometrial cancers that are prevalent in Lynch syndrome families, we oberved an increased risk for individuals whose FDRs were diagnosed CRC after these two cancer, but the association was not significant. This could be due to the small ample size as well as the exlusion of families with multiple affected relatives in the analyses. The latter is supported by the increased CRC risk among individuals who had both FDRs affected by CRCa-2 and FDRs affected by other cancers (Supplementary Table 2, right part). We attempted to quantify the risk for each first primary cancer based on the time between first cancer and CRCa-2 diagnosed in the FDR. Unlike the stable familial risks during the whole period for the overall cancer, fluctuation in different periods was observed for the cancer sites in the Supplementary Table 1. As the number of the cases were small, it is difficult to make any solid conclusion although we discussed first prostate cancer above. Larger studies are needed to investigate when a CRCa-2 is more likely to result in a CRC diagnosis in family member, the results of which could provide evidence on CRC screening in family members.

### Strengths and limitations

By incorporating the Swedish national registers, the order of primary cancer and family relationship can be obtained without selection bias and information bias, thus achieving a validated estimation of familial risk of CRC regarding family history of CRCa-2. Second primary cancers undergo the same rigorous histological diagnostics that are applied for first primary cancers in the Swedish cancer registration system. Based on an ad hoc study, the diagnostic accuracy of second neoplasms was reported to be 98% correctly in the Swedish Cancer Registry; no recorded second primary cancer was found to be a metastasis [23]. We performed a set of stratification analyses, which bridged the knowledge gap on the difference between the family history of CRCa-1 and CRCa-2. We estimated the risk in families with only one FDR affected by either CRCa-1 or CRCa-2 and it is easy to compare the risk among groups. In the supplementary results, we showed that the RRs followed a similar trend as the main analyses. It should be noted that CRC risk among individuals with only one FDR affected by CRCa-2 may be less studied compared to those with multiple primary cancers clustered in a family due to strong familial risk factors. We did not consider the risk among individuals whose FDRs were diagnosed with multiple primary CRC because of the difficulty to differentiate CRCa-2 from CRCa-1. CRC risk factors such as smoking, physical activity and diet are not available in the database, although adjustment for socioeconomic factors and the place of residence can reduce the confounding effect due to their internal correlation [24, 25]. We did not have information on the treatment for the first primary cancer in FDRs, as well as the individual genetic mutation, that could have shed light on the cause of CRCa-2 and interpretation of the familial association. Although this was a nationwide study, we acknowledge that our sample size was not adequate to assess some specific first primary cancers diagnosed in FDRs. Another limitation is that we did not take into account the correlation between individuals within families, and consideration of that could have make the RR estimation more precise. It should be noted that the result observed here may not be generalized to other countries. Sweden has comprehensive healthcare coverage that encompasses the whole population, whereas national screening of CRC doesn’t get implemented with a bias towards a subset of the population. In CRC screening, the precursor lesions will be removed before they become invasive CRC. Consequently, the screening will have some effect on the CRC incidence. The effect on familial risk estimation will depend on the fraction of screening among individuals with and without a family history [26]. More studies among other populations are needed to validate and explain our findings.

### Clinical implication

Although the starting age and the methods for CRC screening vary across countries [27–29], individuals with a family history of CRC were recommended to start the screening at an earlier age. However, no guidance suggests whether family history of CRCa-2 should be equally considered as the family history of CRCa-1. In Sweden, the number of individuals with a family history of CRCa-2 is more than 10% of those with CRCa-1. To the best of our knowledge, this is the first study regarding familial risk related to the family history of CRCa-2. Our results identified an overall lower familial risk for individuals with one FDR affected by CRCa-2 than those with one FDR affected by CRCa-1. The insignificant result for the comparison after adjusting the age at diagnosis of CRC and family relationship indicates these two groups of individuals can be managed similarly, as in the current CRC screening guideline age at diagnosis of CRC in FDRs has been applied to decide the starting age of the CRC screening [27–29]. The current results also imply the limited effect of cancer treatment on familial risk. Therefore, the start age at CRC screening should be considered for those with FDR affected by CRCa-2.

## Conclusions

In this nationwide cohort study, we found that the risk of CRC was significantly increased among individuals with a FDR affected by CRCa-2, but the magnitude was lower than that among individuals with a FDR affected by CRCa-1, which could be related to different ages at diagnosis of CRC in FDR and family relationships. Our results do not support on change of the CRC screening guideline, given that it already considers age at diagnosis of CRC in relatives, although the order of the primary CRC is not differentiated in relatives.

## Supplementary Information

**Additional file 1: Supplementary Table 1.** Colorectal cancer risk among offspring with a family history of CRCa-2 stratified by type of first primary cancer and period between first primary cancer and CRCa-2 in their relatives. **Supplementary Table 2.** Colorectal cancer risk in association with family history of CRCa-1 and CRCa-2 among all the offspring in the registry stratified by if any other FDR was affected by first primary non-CRC cancer. **Supplementary Fig. 1.** Description of family history identification and risk estimation. Parents and siblings were used to define family history. Cases in the offspring generation were used to estimate risk. In this figure, family history is attributed from mothers as example. In Fig. 1a, mother was first diagnosed with cancer A (first primary cancer) at age1 and then CRCa-2 at age2. In Fig. 1b, mother was diagnosed with CRCa-1. In Fig. 1c, no first-degree relatives were diagnosed with any cancer. CRC, colorectal cancer, cancer A, any cancer other than colorectal cancer, CRCa-1, colorectal cancer as a first primary malignancy, CRCa-2, colorectal cancer as a second primary malignancy. **Supplementary Fig. 2.** Flowchart of the population selection and analyses. Higher order primary cancers, such as third, fourth primary cancers, CRC, colorectal cancer, CRCa-1, colorectal cancer as a first primary malignancy, CRCa-2, colorectal cancer as a second primary malignancy, FDR, first-degree relative (parents or siblings).

## Data Availability

The data that support the findings of this study are available from Lund University but restrictions apply to the availability of these data, which were used under license for the current study and so are not publicly available. Any request regarding the data from this study should go to the last author (J.J.).

## Abbreviations

CRC: Colorectal cancer
CRCa-1: Colorectal cancer as a first primary malignancy
CRCa-2: Colorectal cancer as a second primary malignancy
FDR: First-degree relatives
ICD: International Classification of Disease
RR: Rate ratio
95%CI: 95% confidence interval
UAT: Upper aerodigestive tract
NHL: Non-Hodgkin lymphoma
CUP: Cancer of unknown primary
