# Supplementary material for: Colorectal cancer risk in association with colorectal cancer as a second malignancy in relatives: a nationwide cohort study
Source: BMC Cancer. 2022 Aug 18;22:902. doi: 10.1186/s12885-022-10000-z (PMC9389686; doi:10.1186/s12885-022-10000-z)
Supplement: Supplementary file 1 — Additional file 1: Supplementary Table 1. Colorectal cancer risk among offspring with a family history of CRCa-2 stratified by type of first primary cancer and period between first primary cancer and CRCa-2 in their relatives. Supplementary Table 2. Colorectal cancer risk in association with family history of CRCa-1 and CRCa-2 among all the offspring in the registry stratified by if any other FDR was affected by first primary non-CRC cancer. Supplementary Fig. 1. Description of family history identification and risk estimation. Parents and siblings were used to define family history. Cases in the offspring generation were used to estimate risk. In this figure, family history is attributed from mothers as example. In Fig. 1a, mother was first diagnosed with cancer A (first primary cancer) at age1 and then CRCa-2 at age2. In Fig. 1b, mother was diagnosed with CRCa-1. In Fig. 1c, no first-degree relatives were diagnosed with any cancer. CRC, colorectal cancer, cancer A, any cancer other than colorectal cancer, CRCa-1, colorectal cancer as a first primary malignancy, CRCa-2, colorectal cancer as a second primary malignancy. Supplementary Fig. 2. Flowchart of the population selection and analyses. Higher order primary cancers, such as third, fourth primary cancers, CRC, colorectal cancer, CRCa-1, colorectal cancer as a first primary malignancy, CRCa-2, colorectal cancer as a second primary malignancy, FDR, first-degree relative (parents or siblings). [file 12885_2022_10000_MOESM1_ESM.docx]

**Supplementary Table 1.** Colorectal cancer risk among offspring with a family history of CRCa-2 stratified by type of first primary cancer and period between first primary cancer and CRCa-2 in their relatives

| First primary cancer site | **Period between first primary cancer and CRCa-2 in their relatives** | | | | | | | | | | | | | | | | |
| --- | --- | --- | --- | --- | --- | --- | --- | --- | --- | --- | --- | --- | --- | --- | --- | --- | --- |
|  | Q2 (Q1-Q3) month | **< Q1** | | | | **Q1-** **Q2** | | | | **Q2-** **Q3** | | | | **> Q3** | | | |
|  |  | N | RR | 95%CI | | N | RR | 95%CI | | N | RR | 95%CI | | N | RR | 95%CI | |
| UAT | 68 (28-169) | 2 | 1.78 | 0.45 | 7.14 | 2 | 1.78 | 0.44 | 7.11 | 4 | **4.28** | 1.60 | 11.4 | 2 | 1.16 | 0.29 | 4.66 |
| Breast | 18 (15-24) | 12 | **1.99** | 1.13 | 3.50 | 9 | 1.52 | 0.79 | 2.91 | 6 | 1.04 | 0.46 | 2.31 | 13 | **2.08** | 1.21 | 3.58 |
| Prostate | 47 (17-87) | 15 | 1.39 | 0.84 | 2.31 | 19 | **1.97** | 1.26 | 3.49 | 12 | 1.30 | 0.74 | 2.29 | 13 | 1.33 | 0.77 | 2.30 |
| Kidney | 142 (46-215) | 5 | 2.38 | 0.99 | 5.73 | 4 | **3.85** | 1.45 | 10.3 | 0 | - | - | - | 1 | 2.28 | 0.32 | 16.2 |
| Nervous system | 101 (47.5-200) | 4 | **3.52** | 1.32 | 9.37 | 1 | 0.97 | 0.14 | 6.89 | 4 | **4.32** | 1.62 | 11.5 | 2 | 2.35 | 0.59 | 9.40 |
| All | 72 (25-150) | 63 | **1.43** | 1.12 | 1.83 | 57 | **1.43** | 1.10 | 1.86 | 68 | **1.69** | 1.33 | 2.15 | 63 | **1.45** | 1.13 | 1.86 |

UAT, upper aerodigestive tract, CRCa-2, colorectal cancer as a second primary malignancy.

Time between first primary cancer and CRCa-2 in their relatives was calculated for each first primary cancer sites. The risk was estimated for four specific periods based on first (Q1), second (Q2) and third (Q3) quartiles.

| CRC diagnosis in FDRs | If any other FDR was affected by first primary non-CRC cancer | | | | | | | | | |
| --- | --- | --- | --- | --- | --- | --- | --- | --- | --- | --- |
|  | No | | | | | Yes | | | | |
|  | Number of CRC | Person at risk | IR | RR | 95%CI | Number of CRC | Person at risk | IR | RR | 95%CI |
| No CRC | 16,005 | 5,595,074 | 15.7 | Ref. | - | 21,468 | 2,225,379 | 31.1 | Ref. | - |
| One CRCa-1 | 3,096 | 189,798 | 51.4 | 1.75 | 1.68-1.82 | 2,775 | 117,099 | 64.9 | 1.63 | 1.56-1.69 |
| >1 CRCa-1 | 223 | 7292 | 86.7 | 2.22 | 1.95-2.54 | 271 | 4,159 | 167.6 | 3.33 | 2.96-3.76 |
| One CRCa-2 | 295 | 20,220 | 44.3 | 1.58 | 1.40-1.77 | 358 | 14,193 | 68.9 | 1.79 | 1.61-1.98 |
| >1 CRCa-2 | 4 | 82 | 134.8 | 3.16 | 1.19-8.42 | 6 | 44 | 355.7 | 6.45 | 2.90-14.4 |
| CRCa-1 + CRCa-2 | 55 | 1502 | 104.7 | 2.83 | 2.17-3.68 | 49 | 909 | 136.2 | 2.83 | 2.14-3.75 |

**Supplementary Table 2**. Colorectal cancer risk in association with family history of CRCa-1 and CRCa-2 among all the offspring in the registry stratified by if any other FDR was affected by first primary non-CRC cancer

Individuals with family history of multiple CRCs have been removed.

CRC, colorectal cancer, CRCa-1, colorectal cancer as a first primary malignancy, CRCa-2, colorectal cancer as a second primary malignancy, non-CRC cancer, cancer that occurs in other places than colorectum. IR, incidence rate, presented as number of CRC diagnosis per 100,000 person-years.


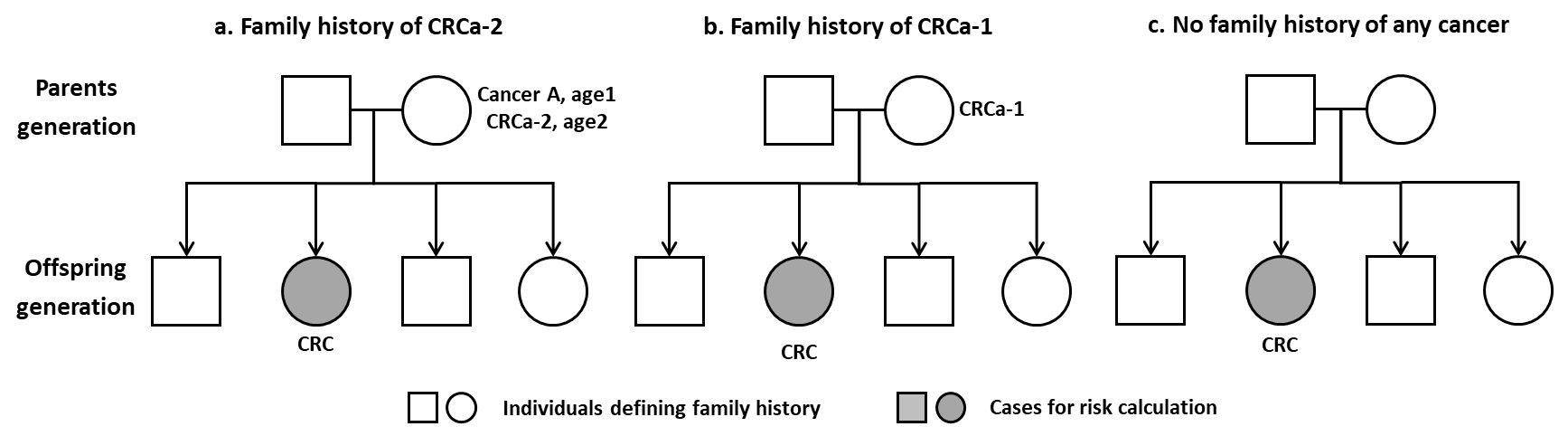


**Supplementary Figure 1.** Description of family history identification and risk estimation. Parents and siblings were used to define family history. Cases in the offspring generation were used to estimate risk. In this figure, family history is attributed from mothers as example. In Figure 1a, mother was first diagnosed with cancer A (first primary cancer) at age1 and then CRCa-2 at age2. In Figure 1b, mother was diagnosed with CRCa-1. In Figure 1c, no first-degree relatives were diagnosed with any cancer. CRC, colorectal cancer, cancer A, any cancer other than colorectal cancer, CRCa-1, colorectal cancer as a first primary malignancy, CRCa-2, colorectal cancer as a second primary malignancy.


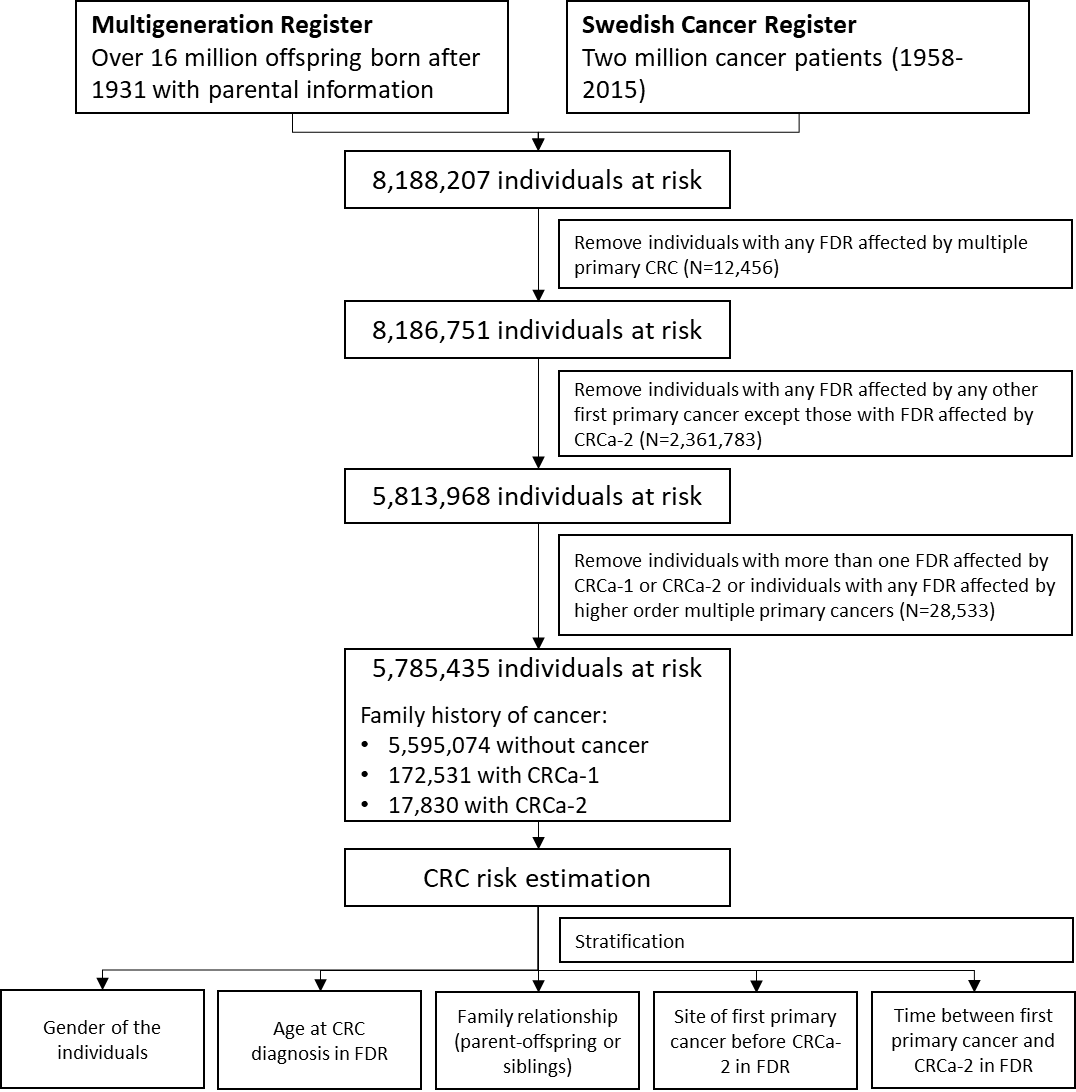


**Supplementary Figure 2**. Flowchart of the population selection and analyses. Higher order primary cancers, such as third, fourth primary cancers, CRC, colorectal cancer, CRCa-1, colorectal cancer as a first primary malignancy, CRCa-2, colorectal cancer as a second primary malignancy, FDR, first-degree relative (parents or siblings).
